# Decreased serum apolipoprotein A1 levels are associated with poor survival and systemic inflammatory response in colorectal cancer

**DOI:** 10.1038/s41598-017-05415-9

**Published:** 2017-07-14

**Authors:** Päivi Sirniö, Juha P. Väyrynen, Kai Klintrup, Jyrki Mäkelä, Markus J. Mäkinen, Tuomo J. Karttunen, Anne Tuomisto

**Affiliations:** 1 Cancer and Translational Medicine Research Unit, University of Oulu, POB 5000, 90014 Oulu, Finland; 2 Oulu University Hospital and Medical Research Center Oulu, POB 21, 90029 Oulu, Finland; 3 Research Unit of Surgery, Anesthesia and Intensive Care, University of Oulu, POB 5000, 90014 Oulu, Finland

## Abstract

Recent studies have reported of an association between high serum apolipoprotein A1 (APOA1) levels and favorable prognosis in several malignancies, while the significance of apolipoprotein B (APOB) in cancer is less well-known. In this study, we analyzed the correlation between serum APOA1 and APOB levels, and APOB/APOA1 ratio, and their associations with clinicopathologic parameters, the levels of twenty systemic inflammatory markers, and survival in 144 colorectal cancer (CRC) patients. We demonstrated that low serum APOA1 levels associated with advanced T-class and TNM-stage but low serum APOB levels did not significantly correlate with tumor characteristics. Serum APOA1 levels showed strong negative correlation with the markers of systemic inflammation including serum CRP and interleukin (IL)-8 levels and blood neutrophil count, whereas high serum APOB levels associated with high serum CCL2 levels. High APOA1 and APOB levels and low APOB/APOA1 ratio associated with improved cancer specific and overall survival. APOA1 had independent prognostic value in Cox regression analysis. In conclusion, low serum APOA1 levels are associated with advanced stage and systemic inflammation, while serum APOB does not significantly correlate with tumor stage. Serum APOA1 represents a promising additional prognostic parameter in CRC.

## Introduction

Apolipoprotein A1 (APOA1), a predominant protein component in high-density lipoprotein (HDL), is mainly synthesized in the liver and in the small intestine. APOA1/HDL particles transport excess cholesterol from peripheral tissues to the liver, while they also have anti-inflammatory, anti-apoptotic and anti-oxidant functions^1^. APOA1 promotes ABCA1-mediated cholesterol and phospholipid efflux which initiates HDL synthesis^2^. It is also a cofactor for lecithin cholesterol acyl transferase (LCAT), an enzyme that converts cholesterol to cholesterol esters^3^. Serum HDL levels are inversely related to the risk of atherosclerosis and cardiovascular disease^4–6^. Low-density lipoprotein (LDL) and its component apolipoprotein B (APOB) transport cholesterol to peripheral tissues, and the APOB/APOA1 ratio represents the balance between proatherogenic and antiatherogenic lipoproteins. Indeed, APOB/APOA1 ratio may represent superior risk indicator of cardiovascular events compared with lipid parameters^7–9^.

Colorectal cancer (CRC) is the second most common cause of cancer-related deaths in the Western world^10^. Abnormal levels of lipids have been linked with cancer risk and progression in several malignancies^11^. However, high serum APOA1 and HDL levels have been associated with a decreased risk of several cancers^12, 13^, as well as premalignant lesions, including colorectal adenomas^14–16^ and CRC^15, 17, 18^. Moreover, decreased serum APOA1 levels in CRC patients have been reported^19, 20^, but to our knowledge, the relationships between serum APOA1 levels and clinicopathological parameters of CRC are unclear. In some malignancies, low serum APOA1 levels have been reported to correlate with poor disease outcome^21, 22^ but the mechanisms underlying this association and the potential prognostic value of serum APOA1 in CRC are not well-known. The association of APOB with cancer is far less explicit, since APOB studies have mostly focused on cardiometabolic disorders. Elevated serum APOB levels have been associated with diabetes and metabolic syndrome^23, 24^, both of which may have effect on cancer development^25, 26^. Moreover, a recent publication revealed associations between high APOB level and increased lung cancer and colorectal cancer risk and an association between low APOB and increased breast cancer risk^11^.

The presence of a systemic inflammatory response (SIR) has been linked with advanced disease and poor survival in CRC^27–30^. SIR, induced by malignancies or other conditions such as infection, inflammation or trauma, is known to result in changes in lipid metabolism^31^. The modified Glasgow Prognostic Score (mGPS), a summary score based on the presence of an increased serum CRP and decreased serum albumin, is a measure of systemic inflammation and has been reported to harbor independent prognostic value in CRC^28^. Inflammatory response is mediated by the release of proinflammatory cytokines, and increased serum levels of interleukin (IL)-1ra, IL-6, IL-7, IL-8, IL-9, IL-12, interferon (IFN)-γ, chemokine, cc motif, ligand (CCL)2, chemokine, cxc motif, ligand 10 (CXCL10), CCL4 and platelet-derived growth factor, subtype BB (PDGF-BB) have been associated with elevated mGPS in CRC patients^27^. However, the relationships between SIR and serum APOA1 and APOB levels have not been thoroughly characterized.

The aim of this study was to investigate the serum APOA1 and APOB levels, and APOB/APOA1 ratio in CRC in relation to clinicopathological factors, with special emphasis on their prognostic significance and their associations with the markers of systemic inflammation.

## Results

### Serum APOA1 levels, serum APOB levels and APOB/APOA1 ratio in relation to clinicopathological characteristics

The characteristics of CRC patients are shown in Table 1. The median serum APOA1 and APOB levels, and APOB/APOA1 ratio were 1.30 g/L, 0.75 g/L and 0.578, respectively. APOA1 levels positively correlated with APOB levels (r = 0.291, *P < *0.001).

**Table 1:** Patient characteristics.

Colorectal cancer patients (n = 144)	
**Age**, **mean (SD)**	66.6 (11.0)
**Gender**	
Male	77 (53.5%)
Female	67 (46.5%)
**Preoperative RT/CRT**	
No	112 (77.8%)
Yes	32 (22.2%)
**Tumor location**	
Proximal colon	49 (34.0%)
Distal colon	27 (18.8%)
Rectum	68 (47.2%)
**WHO grade**	
Grade 1	19 (12.2%)
Grade 2	105 (73.4%)
Grade 3	19 (13.2%)
**TNM stage**	
Stage I	25 (17.4%)
Stage II	54 (37.5%)
Stage III	44 (30.6%)
Stage IV	19 (13.2%)

Abbreviations: SD: standard deviation; RT/CRT: radiotherapy or chemoradiotherapy.

We first analyzed the relationships between serum APOA1 and serum APOB levels and the clinicopathological variables (Table 2). Serum APOA1 levels inversely correlated with TNM stage (*P* = 0.038), especially T-stage (*P* = 0.008), and WHO grade (*P* = 0.024). In addition, women had higher APOA1 levels compared with men (*P* = 0.034). APOB levels showed two significant associations with variables studied: higher APOB levels in younger patients (<65 years) compared with elderly patients (≥65) (*P* = 0.035) and lower APOB levels in cholesterol-lowering medication users compared with non-users (*P* = 0.001). Increased APOB/APOA1 ratio significantly associated with nodal metastases (*P* = 0.010), high tumor necrosis percentage (*P* = 0.041), and decreased APOB/APOA1 ratio with cholesterol-lowering medication (*P* = 0.015).

**Table 2:** Serum apolipoprotein A1 (APOA1) and apolipoprotein B (APOB) levels in relation to clinical and pathological characteristics of tumors

	APOA1 (g/L), median (IQR)	*P* value	APOB (g/L), median (IQR)	*P* value	APOB/APOA1 ratio, median (IQR)	*P* value
**Gender**						
Male (n = 77)	1.283 (1.201–1.390)	**0.034**	0.747 (0.652–0.884)	0.908	0.585 (0.502–0.680)	0.292
Female (n = 67)	1.346 (1.247–1.451)		0.753 (0.651–0.869)		0.562 (0.497–0.657)	
**Age**						
< 65 years (n = 58)	1.285 (1.230–1.435)	0.651	0.781 (0.675–0.928)	**0.035**	0.599 (0.509–0.699)	0.063
≥ 65 years (n = 86)	1.314 (1.231–1.421)		0.729 (0.644–0.826)		0.557 (0.500–0.646)	
**BMI**						
<25 (n = 58)	1.307 (1.221–1.424)	0.084	0.740 (0.651–0.813)	0.487	0.571 (0.506–0.630)	0.287
25–30 (n = 58)	1.314 (1.244–1.476)		0.762 (0.655–0.906)		0.582 (0.491–0.679)	
>30 (n = 26)	1.267 (1.203–1.378)		0.762 (0.647–0.897)		0.597 (0.545–0.665)	
**Tumor location**						
Proximal colon (n = 49)	1.268 (1.223–1.444)	0.184	0.753 (0.655–0.878)	0.241	0.573 (0.490–0.684)	0.964
Distal colon (n = 27)	1.278 (1.073–1.371)		0.688 (0.641–0.797)		0.562 (0.509–0.657)	
Rectum (n = 68)	1.316 (1.248–1.431)		0.766 (0.669–0.896)		0.582 (0.506–0.661)	
**TNM Stage**						
Stage I (n = 25)	1.392 (1.251–1.558)	**0.038**	0.780 (0.691–0.960)	0.282	0.592 (0.517–0.669)	0.202
Stage II (n = 54)	1.332 (1.235–1.421)		0.733 (0.648–0.852)		0.525 (0.495–0.644)	
Stage III (n = 44)	1.303 (1.231–1.445)		0.753 (0.658–0.876)		0.585 (0.523–0.663)	
Stage IV (n = 19)	1.251 (1.073–1.311)		0.768 (0.612–0.957)		0.627 (0.501–0.780)	
**Depth of invasion**						
T1 (n = 4)	1.318 (1.208–1.553)	**0.008**	0.837 (0.666–1.085)	0.084	0.590 (0.549–0.755)	0.653
T2 (n = 28)	1.362 (1.274–1.547)		0.791 (0.670–0.956)		0.593 (0.509–0.677)	
T3 (n = 100)	1.303 (1.232–1.411)		0.746 (0.650–0.876)		0.563 (0.498–0.658)	
T4 (n = 11)	1.087 (0.962–1.243)		0.636 (0.598–0.869)		0.552 (0.517–0.763)	
**Nodal metastasis**						
N0 (n = 83)	1.327 (1.234–1.443)	**0.021**	0.747 (0.660–0.877)	0.380	0.558 (0.497–0.658)	**0.010**
N1 (n = = 35)	1.316 (1.252–1.456)		0.747 (0.644–0.869)		0.562 (0.493–0.621)	
N2 (n = 24)	1.239 (1.160–1.303)		0.789 (0.646–0.966)		0.647 (0.589–0.732)	
**Distant metastasis**						
M0 (n = 124)	1.317 (1.233–1.444)	**0.019**	0.748 (0.661–0.876)	0.922	0.576 (0.501–0.652)	0.176
M1 (n = 19)	1.251 (1.073–1.311)		0.768 (0.612–0.957)		0.627 (0.501–0.780)	
**WHO Grade 1–3**						
Grade 1 (n = 19)	1.404 (1.255–1.563)	**0.024**	0.726 (0.660–0.869)	0.897	0.540 (0.493–0.594)	0.196
Grade 2 (n = 105)	1.302 (1.234–1.404)		0.749 (0.661–0.880)		0.580 (0.502–0.660)	
Grade 3 (n = 19)	1.243 (1.047–1.419)		0.732 (0.598–0.948)		0.622 (0.495–0.761)	
**Necrosis**						
9% or less (n = 71)	1.316 (1.247–1.428)	0.179	0.739 (0.654–0.855)	0.362	0.558 (0.485–0.634)	**0.041**
10% or more (n = 72)	1.294 (1.219–1.429)		0.771 (0.650–0.885)		0.589 (0.517–0.688)	
**Modified Glasgow Prognostic score (mGPS)**						
0 (n = 113)	1.316 (1.242–1.447)	**0.001**	0.753 (0.674–0.882)	0.108	0.574 (0.496–0.658)	0.154
1–2 (n = 31)	1.216 (1.047–1.369)		0.726 (0.607–0.850)		0588 (0.524–0.763)	
**Cholesterol-lowering medication**						
No (n = 98)	1.313 (1.230–1.452)	0.171	0.777 (0.679–0.902)	**0.001**	0.592 (0.515–0.684)	**0.015**
Yes (n = 46)	1.285 (1.231–1.367)		0.711 (0.602–0.762)		0.538 (0.495–0.592)	

Abbreviations: IQR: interquartile range; BMI: body mass index. *P* values are for Mann-Whitney or Kruskal-Wallis test.

### Serum APOA1 levels, serum APOB levels and APOB/APOA1 ratio in relation to systemic inflammatory markers

Next, we investigated the associations between serum APOA1 and serum APOB levels, and their ratio, and systemic inflammation (Table 3). We found that, especially, serum APOA1 concentrations inversely correlated with several markers of systemic inflammation. The strongest correlations were seen between APOA1 and serum CRP (r = −0.436, *P* < 0.001), blood neutrophil count (r = −0.413, *P* < 0.001) and serum IL-8 (r = −0.425, *P* < 0.001). APOA1 also correlated with blood leukocyte count, blood monocyte count and serum IL-1ra, IL-6, IL-7, IL-9, IL-12, CXCL10, CCL4 and PDGF-BB levels. In addition, APOA1 levels were lower in patients with moderate or high mGPS score compared to those with low mGPS (Table 2, *P* = 0.001). Serum APOB levels correlated with serum CCL2 levels (r = 0.223, *P* = 0.007) and negatively correlated with blood neutrophil count (r = −0.178, *P* = 0.033). APOB/APOA1 ratio correlated positively with serum levels of several cytokines (IL-1ra, IL-6, IL-7, IL-8, IFNγ, CCL2 and PDGF-BB).

**Table 3:** Correlations between serum APOA1, APOB and systemic inflammatory markers.

	^a^mg/L, ^b^x 10^9^, or ^c^pg/mL, median (IQR)	APOA1		APOB		APOB/APOA1 ratio	
		Pearson r	*P* value	Pearson r	*P* value	Pearson r	*P* value
Serum C-reactive protein	2.22 (0.71–8.17)^a^	−0.436	**<0.001**	−0.156	0.062	0.123	0.141
Blood leukocyte count	6.70 (5.20–7.90)^b^	−0.396	**<0.001**	−0.138	0.099	0.116	0.168
Blood neutrophil count	4.00 (2.91–5.14)^b^	−0.413	**<0.001**	−0.178	**0.033**	0.087	0.299
Blood lymphocyte count	1.70 (1.20–2.27)^b^	−0.1125	0.137	−0.035	0.675	0.045	0.597
Blood monocyte count	0.60 (0.40–0.70)^b^	−0.302	**<0.001**	−0.127	0.131	0.067	0.426
Blood neutrophil/lymphocyte ratio	2.38 (1.91–3.29)	−0.218	**<0.001**	−0.111	0.189	0.030	0.723
Serum IL-1ra	62.9 (37.9–94.5)^c^	−0.352	**<0.001**	0.007	0.935	0.230	**0.006**
Serum IL-4	0.86 (0.72–1.12)^c^	−0.128	0.126	0.075	0.372	0.155	0.064
Serum IL-6	4.81 (3.41–7.94)^c^	−0.394	**<0.001**	0.038	0.648	0.288	**<0.001**
Serum IL-7	5.73 (4.31–7.73)^c^	−0.240	**0.004**	0.050	0.550	0.201	**0.016**
Serum IL-8	11.8 (8.99–16.90)^c^	−0.425	**<0.001**	0.085	0.309	0.353	**<0.001**
Serum IL-9	8.59 (5.91–13.50)^c^	−0.185	**0.027**	0.021	0.805	0.138	0.101
Serum IL-12(p70)	29.0 (13.5–40.4)^c^	−0.214	**0.010**	−0.150	0.073	−0.011	0.896
Serum IFNγ	30.5 (23.6–42.8)^c^	−0.143	0.086	0.094	0.262	0.183	**0.028**
Serum CXCL10	903.3 (680.7–1215.2)^c^	−0.268	**0.001**	−0.029	0.729	0.141	0.092
Serum CCL2	17.9 (12.2–27.0)^c^	−0.124	0.142	0.223	**0.007**	0.297	**<0.001**
Serum CCL4	65.3 (51.0–84.0)^c^	−0.247	**0.003**	−0.063	0.455	0.095	0.258
Serum CCL11	131.2 (90.7–180.4)^c^	0.014	0.869	0.021	0.802	0.012	0.888
Serum PDGF-BB	8433.5 (5875.0–11324.0)^c^	−0.210	**0.011**	0.047	0.579	0.179	**0.032**

Abbreviations: IL: interleukin; IFN: interferon; CCL: chemokine (C-C motif) ligand; PDGF: platelet-derived growth factor. Numbers indicate Pearson correlation coefficients (r) for logarithmically transformed variables.

### Survival analyses

**Figure 1:**
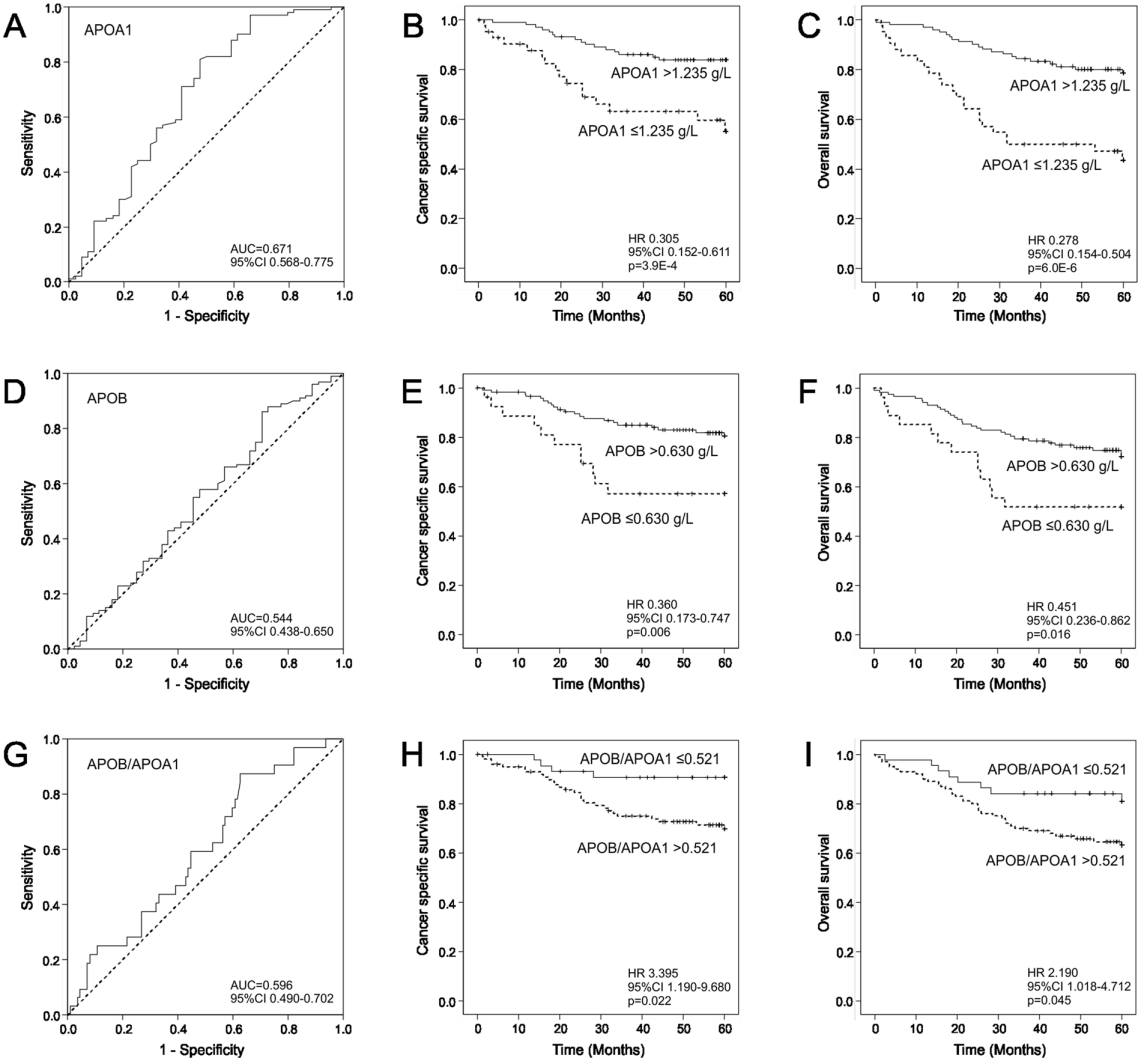
Survival analyses. (**A**) Receiver operating characteristics (ROC) curve for serum APOA1 in predicting overall survival (OS). Kaplan-Meier curves show that higher serum APOA1 level associates with better cancer-specific survival (CSS) (**B**) and overall survival (**C**). ROC curve for serum APOB in predicting OS (**D**). Serum APOB level and CSS (**E**). Serum APOB level and OS (**F**). ROC curve for serum APOB/APOA1 ratio in predicting OS (**G**). Serum APOB/APOA1 ratio and CSS (**H**). Serum APOB/APOA1 level and OS (**I**).

Finally, we investigated the prognostic significance of serum APOA1 and APOB levels, and APOB/APOA1 ratio in CRC. The optimal cutoff points were based on the ROC analyses (APOA1: overall survival (OS), area under the curve: 0.671, 95% confidence interval (CI): 0.568–0.775, *P* = 0.001, Fig. 1A; APOB: OS, area under the curve:0.544, 95% CI: 0.438–0.650, *P* = 0.403, Fig. 1D; APOB/APOA1 ratio OS, area under the curve: 0.596, 95% CI: 0.490–0.702, *P* = 0.099, Fig. 1G), and the patients were divided into high and low serum APOA1, APOB and APOB/APOA1 ratio groups (cut-off points 1.235 g/L, 0.630 g/L, and 0.521, respectively).

Kaplan-Meier curves visualized that high serum APOA1 levels significantly associated with better cancer-specific survival (CSS) (61.9% vs. 84.3%, *P* =  < 0.001, Fig. 1B) and OS (45.2% vs. 79.4%, *P =  < *0.001, Fig. 1C). Similarly, high APOB levels associated with better CSS (59.3% vs 82.1%, *P* = 0.006, Fig. 1E) and OS (51.9% vs 73.5%, *P* = 0.016, Fig. 1F) and low APOB/APOA1 ratio showed association with better CSS (90.9% vs 72.0%, *P* = 0.022, Fig. 1H) and OS (81.8% vs 64.0%, *P* = 0.045, Fig. 1I).

The analyses indicated that the prognostic significance of APOA1 was higher than APOB. Due to high intercorrelation between these variables, only APOA1 was included in the final Cox regression model. In that model, increased serum APOA1 associated with better CSS and OS independent of other clinicopathological variables, including mGPS, a validated systemic inflammation-based prognostic parameter (Table 4). In addition to serum APOA1, patient age, N-stage, and M-stage remained statistically significant in the model.

**Table 4:** Multivariate analysis of 60 month cancer-specific survival (CSS) and overall survival (OS) of CRC patients.

	CSS			OS		
	HR	95%CI	*P* value	HR	95%CI	*P* value
Age (**<**65 vs.≥65)	3.31	1.34–8.15	**0.009**	2.48	1.20–5.13	**0.015**
Tumor invasion (T1-T2 vs. T3-T4)	1.46	0.39–5.45	0.571	1.25	0.48–3.30	0.649
Nodal metastases (N0 vs. N1-N2)	4.94	2.02–12.12	**<0.001**	2.84	1.44–5.61	**0.003**
Distant metastases (M0 vs. M1)	4.73	1.81–12.32	**0.001**	3.00	1.33–6.74	**0.008**
Tumor location (colon vs. rectum)	2.01	0.77–5.29	0.157	1.64	0.73–3.67	0.232
Preoperative radiotherapy or chemoradiotherapy (no vs. yes)	0.31	0.08–1.21	0.092	0.379	0.13–1.12	0.079
mGPS (0 vs. 1–2)	1.20	0.47–3.07	0.699	1.24	0.58–2.69	0.580
Serum APOA1 (≤1.235 vs.>1.235 g/l)	0.37	0.17–0.81	**0.012**	0.318	0.17–0.60	**<0.001**

Abbreviations: CI: confidence interval; HR: hazard ratio.

## Discussion

In this study, we assessed the serum APOA1 and APOB levels and APOB/APOA1 ratio in relation to clinicopathological parameters, patient survival, and systemic inflammation in CRC. We found that low serum APOA1 levels associated with higher stage, especially higher T-class, the presence of systemic inflammatory response, and worse patient outcome. Serum APOB levels did not show significant associations with tumor parameters, but positively correlated with serum APOA1 and CCL2 levels. Increased APOB/APOA1 ratio associated with nodal metastases, abundant necrosis, and serum levels of several cytokines.

Tumor-induced systemic inflammation represents an important regulator of cancer progression and metastasis^32^. About 20% of the CRC patients have elevated serum CRP levels^29^, and increased CRP and mGPS, combined evaluation of serum CRP and albumin, have been linked with adverse clinical outcome and represent promising additional prognostic indicators in CRC^28^. The results of this study indicate that serum APOA1 levels are closely associated with systemic inflammation in CRC. Indeed, APOA1 is a negative acute-phase protein, *i.e*., its expression is lowered more than 25% during the acute-phase response^33^. Although serum APOA1 levels had high correlation with serum APOB levels, APOB levels only associated with serum CCL2 levels, but not with other twelve cytokines analyzed nor with mGPS. These findings suggest that systemic inflammation has less impact on serum APOB than APOA1 in CRC. Conversely, previous studies have detected positive correlation between APOB and inflammatory markers in postmenopausal, overweight women (CRP and IL-6)^34^ and in healthy subjects (CRP)^35^. This suggests that in CRC, APOB homeostasis is less related to SIR than in healthy general population, the causes of which remain unknown.

Systemic inflammation is also known to induce direct changes on HDL particle and APOA1 molecule concentrations. For example, serum amyloid A (SAA) expression is markedly increased in response to acute and chronic inflammation^36^, and circulating SAA is mainly transported on HDL. The presence of SAA on HDL along with other SIR-induced changes on HDL, such as modifications of APOA1 molecule, including oxidation^37^, could render anti-inflammatory HDL into a pro-inflammatory particle. The potential functional consequences and prognostic value of such modifications in CRC represents important subjects for further research.

This study demonstrates novel data on the relationships between tumor characteristics in CRC and serum APOA1 and APOB levels and APOB/APOA1 ratio. Specifically, we found negative correlations between serum APOA1 levels and tumor stage, especially T-class, and WHO grade. We hypothesize that these associations may reflect the strong negative correlation between APOA1 and systemic inflammatory markers, since we and others have previously reported that systemic inflammation is associated with high tumor stage and poor tumor differentiation in CRC^27, 29^. Instead, serum APOB levels or APOB/APOA1 ratio did not significantly associate with tumor stage, suggesting that tumor progression has less impact on these parameters. Patient age or gender did not influence serum APOA1 levels. However, serum APOB levels were lower in older patients but the cause or the significance of this finding is not clear.

Currently, the prognostic classification of CRC is mostly based on TNM staging, but additional prognostic or predictive parameters would be valuable. Our results indicate that lower serum APOA1 levels, lower serum APOB levels, and high APOB/APOA1 ratio are associated with adverse CSS and OS. Of these parameters, serum APOA1 had independent prognostic significance in the Cox regression model. Earlier, circulating APOA1 levels have been reported to associate with poor survival in several other malignancies^21, 22^ but the underlining mechanisms have not been completely understood. Our study suggests that systemic inflammation could be one of the factors explaining the role of APOA1 in survival. However, in our data, the association between decreased APOA1 and adverse outcome was independent of mGPS, an established systemic inflammation based prognostic marker^28^, suggesting that also other factors may be involved. Furthermore, this result encourages subsequent larger-scale studies to evaluate the potential of serum APOA1 as an additional prognostic indicator in CRC, especially in relation to systemic inflammation based markers. Potential confounding of the prognostic value of APOA1 by systemic inflammation should also be taken account when studying the significance of APOA1 in other malignancies.

We have previously shown that tumor necrosis in CRC is related to advanced tumor stage and adverse prognosis but the biological mechanisms of tumor necrosis are largely unknown^38^. In the current study, extensive tumor necrosis was associated with a high APOB/APOA1 ratio, which could, at least partly, be related to the association between tumor necrosis and systemic inflammation^39^. However, since high APOB/APOA1 ratio is considered atherogenic^40^, this encourages further studies to assess, whether atherogenic factors contributes to tumor necrosis in CRC. For renal carcinomas association between tumor necrosis and atherosclerotic changes of renal artery has already been observed^41^.

The strengths of the study were the prospectively recruited, well characterized study cohort and relatively long follow-up period. Moreover, an extensive set of different markers of systemic inflammation were analyzed. The limitations included the lack of data on the patients’ diet and lifestyle, which could have an effect on serum APOA1 and APOB levels^42^, as well as limited sample size. Therefore, further studies are required to establish the potential clinical utility of APOA1 as a prognostic marker of CRC.

In conclusion, we showed that decreased serum APOA1 levels associate with systemic inflammation and adverse survival in CRC. The coincidence of low APOA1 levels and the upregulation of the systemic inflammatory response warrants further studies on their functional relationship and relative prognostic significance in CRC and in other malignancies.

## Methods

### Patients

This prospective study was introduced to all newly diagnosed CRC patients operated in the Oulu University Hospital between April 2006 and January 2010 (n = 344). Preoperative blood samples and surgical specimens were originally collected from 149 patients, who had signed an informed consent to participate and were eligible to the study^27^. The patients with earlier or simultaneously diagnosed other malignant diseases were excluded. Five of 149 (3.4%) cases were not applicable to this study due to insufficient sample material. Clinical data was collected from the clinical records and a questionnaire. The 60-month follow-up data was acquired from the clinical records and Statistics Finland^43, 44^. The study was conducted under the acceptance of the Ethical Committee of the Oulu University Hospital (58/2005, 184/2009) and according to the principles of the Declaration of Helsinki.

### Histopathological analysis

The tumors were staged according to TNM6^45^ and graded according to the WHO criteria^46^. The percentage of tumor tissue showing coagulative necrosis was evaluated by visually inspecting all the available hematoxylin and eosin stained tumor sections^38^. Tumor necrosis was specified as an area with increased eosinophilia and nuclear shrinkage, fragmentation and disappearance, with shadows of tumor cells visible to variable extent^38^.

### Analysis of blood samples

Preoperative serum samples were collected in tubes without clot activator. The samples were centrifuged and stored at −70 °C until the analysis. Nuclear magnetic resonance metabolomics platform, equipped with Bruker AVANCE III 500 MHz and Bruker AVANCE III 600 MHz spectrometers (Bruker, Billerica, MA, USA), was used to analyze serum APOA1 and APOB levels. The analysis process has been outlined previously in more detail^47^. Serum levels of 13 cytokines were analyzed with Bio-Plex Pro Human pre-manufactured 27-Plex Cytokine Panel (Bio-Rad, Hercules, CA, USA)^27^. Blood leukocyte, neutrophil, monocyte and lymphocyte counts, serum CRP levels and serum albumin levels were measured in the laboratory of Oulu University Hospital and mGPS was calculated from serum CRP and albumin values^27, 48^.

### Statistical analyses

Statistical analyses were performed using IBM SPSS Statistics for Windows version 22.0 (IBM Corporation, Armonk, NY, USA). Normally distributed continuous variables are presented as mean (standard deviation, SD), whereas other continuous variables are presented as median (interquartile range, IQR). Correlations between two continuous variables were presented as Pearson correlation coefficients (r). Statistical significances of the differences in serum APOA1, APOB and APOB/APOA1 ratio between the different study groups and categorical variables were analyzed by Mann-Whitney *U* test or Kruskal-Wallis test. ROC analysis was used to determine an optimal cutoff point for serum APOA1, APOB and APOB/APOA1 ratio in detecting patients, who survived in 60 month follow-up. Kaplan-Meier method, Log rank test, and multivariate Cox regression model were used in the survival analyses. A two-tailed *P* < 0.05 was considered statistically significant.

### Data availability statement

All data generated or analyzed during this study are available from the corresponding author on reasonable request.
